# *Nor*-Lignans: Occurrence in Plants and Biological Activities—A Review

**DOI:** 10.3390/molecules25010197

**Published:** 2020-01-03

**Authors:** Claudio Frezza, Alessandro Venditti, Chiara Toniolo, Daniela De Vita, Marco Franceschin, Antonio Ventrone, Lamberto Tomassini, Sebastiano Foddai, Marcella Guiso, Marcello Nicoletti, Mauro Serafini, Armandodoriano Bianco

**Affiliations:** 1 Department of Environmental Biology, University of Rome “La Sapienza”, Piazzale Aldo Moro 5, 00185 Rome, Italy; chiara.toniolo@uniroma1.it (C.T.); daniela.devita@uniroma1.it (D.D.V.); antonio.ventrone@uniroma1.it (A.V.); lamberto.tomassini@uniroma1.it (L.T.); sebastiano.foddai@uniroma1.it (S.F.); marcello.nicoletti@uniroma1.it (M.N.); mauro.serafini@uniroma1.it (M.S.); 2 Department of Chemistry, University of Rome “La Sapienza”, Piazzale Aldo Moro 5, 00185 Rome, Italy; marco.franceschin@uniroma1.it (M.F.); marcella.guiso@uniroma1.it (M.G.)

**Keywords:** *nor*-lignans, occurrence, biological activities

## Abstract

In this review article, the occurrence of *nor*-lignans and their biological activities are explored and described. *Nor*-lignans have proven to be present in several different families also belonging to chemosystematically distant orders as well as to have many different beneficial pharmacological activities. This review article represents the first one on this argument and is thought to give a first overview on these compounds with the hope that their study may continue and increase, after this.

## 1. Introduction

A large part of secondary plant phenolic natural products derives from the aromatic amino acids couple tyrosine/phenylalanine. The de-amination of these compounds gives raise, through the shikimic acid pathway, to the intermediate metabolites C(6)–C(3), i.e., propenyl-phenols and allyl-phenols, generally named as phenylpropanoids. These are the starting points of the biosynthesis of several classes of active constituents related to the stability of the cell wall and to the defense of plants against herbivorous animals and pathogens. The first line of defense in terrestrial plants is a mechanical one, related to a polymerization process which leads to the formation of lignin, the main component of wood. Lignin is a very strong and stable macromolecule. Its introduction inside the plant cell wall instead of the carbohydrate polymer cellulose, confers force and resistance, allowing the formation of giant tree’s structure and making the digestions of the adult parts of the plant from herbivorous animals very difficult. Yet, the lignin defense line has resulted to be quite insufficient in many cases and the incoming predominance of herbal species determined the shift towards another form of defense, i.e., the chemical one. Actually, the phenylpropanoids pathway has never been dismissed, but rather it has turned towards the synthesis of smaller products having more precise targets. Among these molecules, the dimerization process of C(6)–C(3) precursors gives rise to three important classes of natural secondary metabolites: lignans, *neo*-lignans and *nor*-lignans. These classes present similar features due to their common biosynthetic origin. Their general structure is characterized by the presence of two terminal phenyl groups, which are more or less functionalized with hydroxyl groups and connected by a central chain of six carbon atoms, differently arranged and oxidized. The main difference among lignans, *neo*-lignans and *nor*-lignans is due to the different type of junction between the two C(6)C(3) (=PhC3) units. In particular, in lignans, this junction is through a β-β (8-8′) bond and in *neo*-lignans the junction is not a β-β type. Therefore, lignans and *neo*-lignans, and their several different derived subclasses, can be identified depending upon the carbon skeletons which they possess. For what concerns *nor*-lignans, the structure is more complicated. In fact, *nor*-lignans own a peculiar characteristic, with respect to lignans and *neo*-lignans, which is the cut of one carbon from the central chain. This loss forces this chain to be differently arranged from lignans and *neo*-lignans, such as in a linear sequence or in a C(3)C(2) arrangement meaning 8,9′-coupling and 7′,8-coupling or alternatively in the bis-*nor*-lignan and cyclo-*nor*-lignan skeletons (8,8′) where chirality plays a central role. From this description, it is quite easy to understand the other definition of the structure of *nor*-lignans: natural compounds based on diphenyl-pentanes, derived by the union of two phenylpropanoid units in the positions α, β′ or β, γ′ and characterized with the loss of the terminal carbon of the chain [1,2,3].

**Figure 1:**
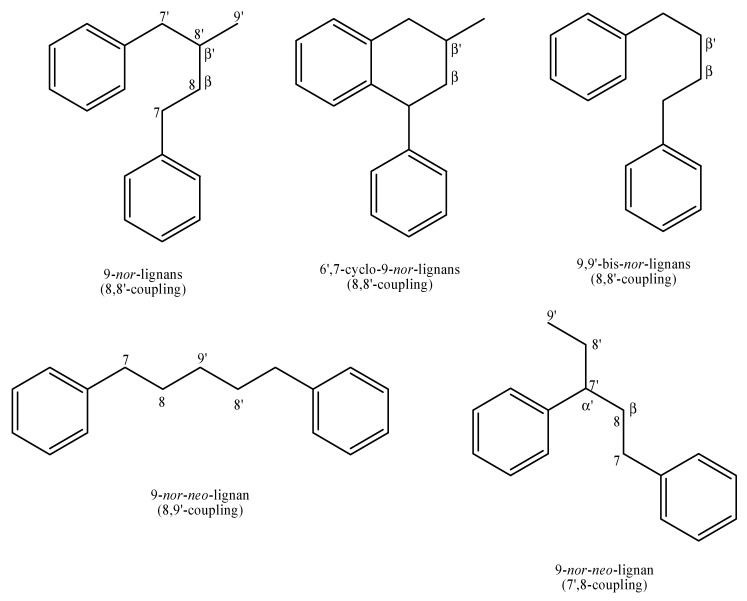
general *nor*-lignan basic structures.

Figure 1 shows the possible different arrangements for *nor*-lignans.

## 2. Occurrence of *Nor*-Lignans in the Plant Kingdom

From the environmental and taxonomical points of view, lignans are mainly biosynthesized in woody plants, since main occurrences are related to Gymnospermae and Angiospermae. In particular, they can be found in the trees’ members of ancient forests like the Amazonian one, but, probably because of their simple biosynthetic pathway, they can be present also in herbal plants like those of monocotyledons.

In this review article, the attention is focused on *nor*-lignans, their occurrence in the plant kingdom and their importance as bioactive molecules.

Table 1 reports on the *nor*-lignans identified in the plant kingdom differentiating them according to the species, genus and family. In addition, the organs from which these compounds have been isolated, and the techniques used for their isolation and identification were completely added.

Figure 2, Figure 3, Figure 4, Figure 5, Figure 6, Figure 7, Figure 8, Figure 9, Figure 10, Figure 11, Figure 12, Figure 13, Figure 14, Figure 15, Figure 16, Figure 17, Figure 18, Figure 19, Figure 20, Figure 21, Figure 22, Figure 23 and Figure 24 below show the structures of all the identified *nor*-lignans.

## 3. Chemotaxonomy

As Table 1 clearly shows, *nor*-lignans have been recognized as phytochemical constituents of several families, even chemosystematically far away from each other.

This is in accordance with the easy phytochemical pathway connected with very common PhC3 intermediate metabolites. However, the rearrangements following the junction of the two originating moieties are another matter.

Therefore, some specific compounds can be evidenced as chemotaxonomic makers at every classification level.

In particular, (*+*)-acortatarinowins A-C, (−)-acortatarinowins A-C (Figure 8) and acorusin B (Figure 15) may be useful chemotaxonomic markers for the species *Acorus tatarinowii* Schott. since they have been isolated only from that species [5,6].

Pachypostaudins A-B and pachypophyllin (Figure 16 and Figure 17) may be chemotaxonomic markers for the entire Annonaceae family given their specific occurrence here [7,8].

Asparenydiol (Figure 17) and its derivatives are considered as some of the chemotaxonomic markers for the genus *Asparagus* L. [17].

Capituloside (Figure 4) and the crassifosides (Figure 10 and Figure 11) may be used as chemotaxonomic markers for the genus *Curculigo* Gaertn. given their occurrence limited to only it [40,43,44,46,51,52].

For the same reason, hypoxoside and related compounds (Figure 12) are a possible chemotaxonomic marker for the genera *Hypoxis* L. and *Curculigo* Gaertner [54,56,59] whereas rataniaphenols I-II (Figure 22) may serve as chemotaxonomic markers for the genus *Krameria* L. [62,63,64].

Within the Lamiaceae family, surely negundins A–B (Figure 20) are chemotaxonomic markers for the species *Vitex negundo* L given their occurrence in several exemplars of this species [69,70,71].

Indeed, egonol, homoegonol and their derivatives (Figure 3) can serve as chemotaxonomic markers for the *Styrax* L. genus since their occurrence is quite limited to it [105,106,107,108,109,110,111].

## 4. Biological Activities

*Nor*-lignans show several interesting biological activities, i.e., antioxidant, antifungal, antibacterial, antiallergic, antiasthma, analgesic, anticomplement, antiatherogenic, antiparasitic, vascular, antistress, anti-inflammatory, cytotoxic, phytotoxic, inhibitory of enzymes, proteins and platelet aggregation. In the following pages, these are characterized one by one.

### 4.1. Antioxidant

Egonol (Figure 3) highly inhibits the production of NO and highly reduces the release of ROS in a dose dependent manner. The same is valid for homoegonol but in a minor way [111].

Indeed, curcapital, crassifogenin C (Figure 9), crassifoside E and crassifoside F (Figure 10) show strong radical scavenging activity by the 1,1-diphenyl-2-picrylhydrazyl (DPPH^•^) assay with IC_50_ values equal to 7.76, 13.48, 15.54 and 17.07 μM, respectively, which are much higher than the control, L-ascorbic acid (IC_50_ = 27.59 μM) [44].

Moreover, hypoxoside and rooperol (Figure 12) show high effects towards the inhibition of lipid peroxidation withIC_50_ values equal to 12.6 and 2.6 μM, respectively [54].

Nyasol (Figure 7) exerts medium effects against ABTS^•+^ cation and superoxide anion radicals with IC_50_ values equal to 45.6 and 40.5 μM, respectively [82].

Vitexdoin F, vitedoin A, 6-hydroxy-4-(4-hydroxy-3-methoxyphenyl)-3-hydroxymethyl-7-methoxy-3,4-dihydro-2-naphthaldehyde, vitexdoin A, negundin B, vitexdoin E, vitrofolal F, 1,2-dihydro-7-hydroxy-1-(4-hydroxy-3-methoxyphenyl)-3-(hydroxymethyl)-6-methoxy-(1*S*,2*R*)-2-naphthalenecarboxaldehyde, vitexdoin C, vitexdoin D, vitrofolal E, vitexdoin B, vitrofolal A and detetrahydro-conidendrin (Figure 20) showed stronger effects than ascorbic acid [69,73].

### 4.2. Antiradical

**Figure 2:**
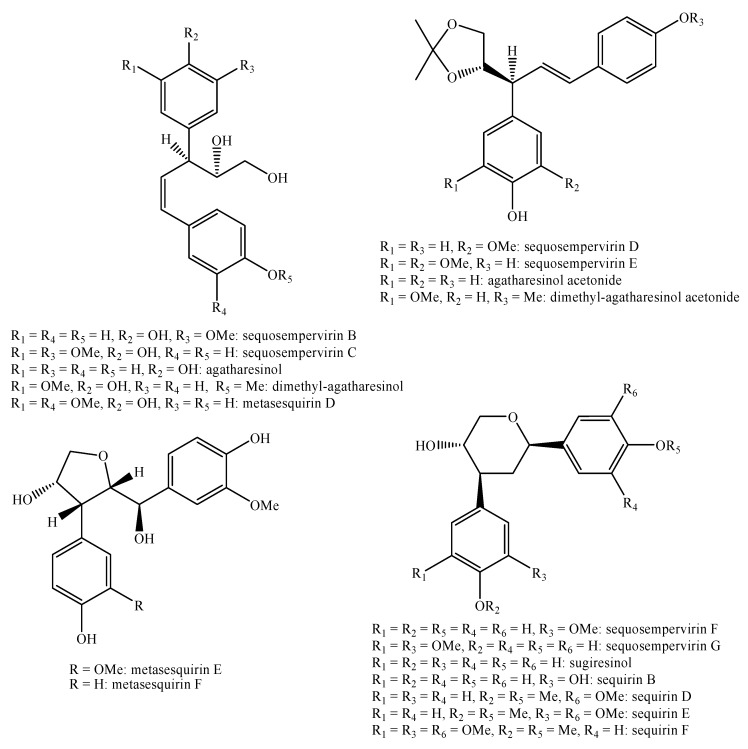
The isolated *nor*-lignans in the plant kingdom—part 1.

Vitrofolal E, vitrofolal F, viteodin A, 6-hydroxy-4-(4-hydroxy-3-methoxyphenyl)-3-hydroxymethyl-7-methoxy-3,4-dihydro-2-naphthaldehyde (Figure 20), detetrahydro-conidendrin (Figure 23) and 2α,3β-7-*O*-methyl-cedrusin (Figure 21) exert high effects against the stable free radical, 1,1-diphenyl-2-picrylhydrazyl (DPPH^•^), more than L-cysteine and, in most cases, similar to α-tocopherol [73].

Vitexnegheteroin E, vitexnegheteroin F, vitexnegheteroin G, vitecannaside B and vitexdoin A (Figure 20) also exhibit strong effects in the ABTS^•+^ assay with IC_50_ values lower than 3.20 μM [74].

Vitexdoin A, vitexdoin B, vitexdoin C, vitexdoin D, vitexdoin E, vitrofolal E and vitrofolal F (Figure 20) are potent NO production inhibitors with IC_50_ values equal to 0.38 μM, 0.20 μM, 0.57 μM, 0.13 μM, 0.15 μM, 0.50 μM and 0.11 μM, respectively.

Instead, 6-hydroxy-4-(4-hydroxy-3-methoxyphenyl)-3-hydroxymethyl-7-methoxy-3,4-dihydro- 2-naphthaldehyde (Figure 20) has a weaker effect with an IC_50_ value equal to 3.54 μM. Anyway, they were all more powerful than the positive control L-nitroarginine (IC_50_= 43.6 μM) [75].

### 4.3. Antifungal and Antibacterial

**Figure 3:**
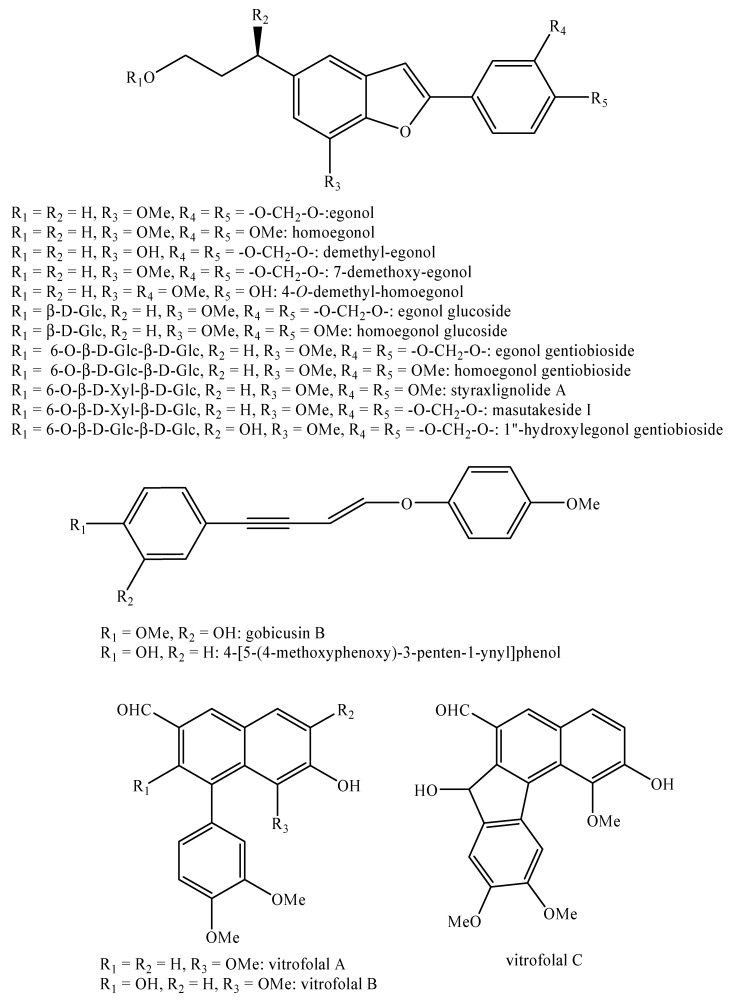
Isolated *nor*-lignans in the plant kingdom—part 2.

Homoegonol and egonol (Figure 3) exhibit strong effects against *Candida albicans*, *Cladosporium sphaerospermum* and *Staphylococcus aureus* with MIC values equal to 10, 5 and 10 μg/mL, respectively for the former compound, and 12, 10 and 10 μg/mL respectively for the latter compound. Indeed, egonol (Figure 3) and homoegonol (Figure 3) exhibit lower effects only against *Candida albicans* and *Staphylococcus aureus* with MIC values equal to 15 and 15 μg/mL, respectively for the former compound and 20 and 20 μg/mL, respectively for the latter compound [106].

Conversely, homoegonol (Figure 3) is totally inactive against *Streptococcus pneumoniae*, *Streptococcus pyogenes*, *Haemophilus influenza*, *Pseudomonas aeruginosa* and *Klebsiella pneumonia* showing MIC values higher than 400 μg/mL. Instead, egonol (Figure 3) is weakly active only against *Streptococcus pneumonia* showing a MIC value equal to 400 μg/mL [115].

*Iso*-agatharesinol and gobicusin A (Figure 7) are also able to exert these effects. In particular, gobicusin A is a better antibacterial compound against *Escherichia coli* and *Staphylococcus aureus* than *iso*-agatharesinol given its MIC values (0.12 and 0.05 mg/mL vs. 0.25 and 0.12 mg/mL, respectively) and its efficacy is extremely comparable to streptomycin especially against *Staphylococcus aureus* (MIC = 0.01 mg/mL) [19].

Nyasol (Figure 7) is able to inhibit the mycelial growth of *Colletotrichum orbiculare*, *Phytophthora capsici*, *Pythium ultimum*, *Rhizoctonia solani* and *Cladosporium cucumerinum* in a MIC range comprised between 1 and 50mg/mL [13]. Moreover, it potently inhibits the growth of *Leishmania major* with an IC_50_ value equal to 12 μM and moderately inhibits *Plasmodium falciparum* with an IC_50_ value equal to 49 μM [14].

Vitrofolal C (Figure 3), vitrofolal D, vitrofolal E (Figure 20) and detetrahydro-conidendrin (Figure 23) have good activity against methicillin-resistant *Staphylococcus aureus* with a MIC value below 64 μg/mL [77].

### 4.4. Antiviral

Nicotnorlignan A, benzodioxane (Figure 19) and sequirin C (Figure 7) showed high effects against HIV-1 with IC_50_ values equal to 3.15, 7.62 and 9.56 μM, respectively [103].

Moreover, nicotnorlignan C, recurphenol C, recurphenol D, benzodioxane (Figure 19) and sequirin C (Figure 7) and possess moderate activity against the anti-tobacco mosaic virus with inhibition rates equal to 14.7%, 22.5%, 23.4%, 21.4% and 17.6% respectively [103]. Nicotnorlignan A (Figure 19) also shows similar effects [103].

### 4.5. Anti-Allergic

Nyasol and 4′-*O*-methyl-nyasol (Figure 7) exert good effects with IC_50_ values equal to 2.06 and 1.89 μM, respectively. These values are extremely compatible with that of DSCG (IC_50_ = 1.78 μM), a very common antiallergic compound used in pharmacy [10].

### 4.6. Antiasthma

Homoegonol (Figure 3) is the only compound able to exert antiasthma effects by a complex mechanism of action composed by several paths [116]. The most important of these is that this compound is able to reduce the expression of the protease MMP-9 in the lung tissue and the presence of this protease greatly increases the asthmatic effect [117].

### 4.7. Analgesic

Hypoxoside (Figure 12) does not display any effect on the locomotor activity in mice but exerts a high analgesic effect even at low doses (5 mg/kg) probably via an anti-inflammatory mechanism [59].

### 4.8. Anticomplement

Styraxlignolide A, egonol and masutakeside I (Figure 3) show a strong effect with IC_50_ values equal to 123, 33 and 166 μM, respectively. This activity, in the case of egonol (Figure 3), is much higher than the control, i.e., rosmarinic acid, which shows an IC_50_ value equal to 182 μM [107].

### 4.9. Antiatherogenic

Nyasol (Figure 7) is able to act as inhibitor against LDL-oxidation with an IC_50_ value equal to 5.6 μM, which is very similar to that of probucol (IC_50_ = 2.0 μM), the typical compound uses for these purposes. Indeed, it exerts extremely weak inhibitory effects against hACAT1, hACAT2 (cholesterol acyltransferases) and Lp-PLA2 (lipoprotein-associated phospholipase A2) with IC_50_ values equal to 280.6, 398.9 and 284.7 μM, respectively [82].

### 4.10. Antiparasitic

3′-methoxy-3,4-methylenedioxy-4′,7-epoxy-9-nor-8,5′-neolignan-9′-acetoxy (Figure 5) has a medium effect against *Trypanosoma cruzi* with an IC_50_ value equal to 111 μM whereas 3′-methoxy-3,4-methylenedioxy-4′,7-epoxy-9-nor-8,5′-neolignan-7,8′-diene (Figure 5) is a good compound in this context with an IC_50_ value equal to 60 μM [78].

### 4.11. Vascular

**Figure 4:**
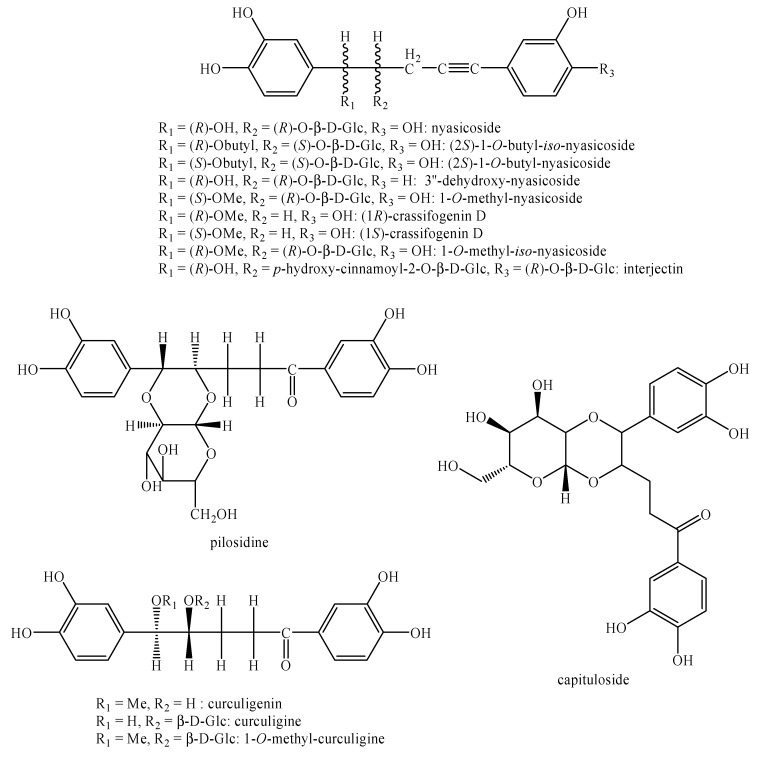
Isolated *nor*-lignans in the plant kingdom—part 3.

**Figure 5:**
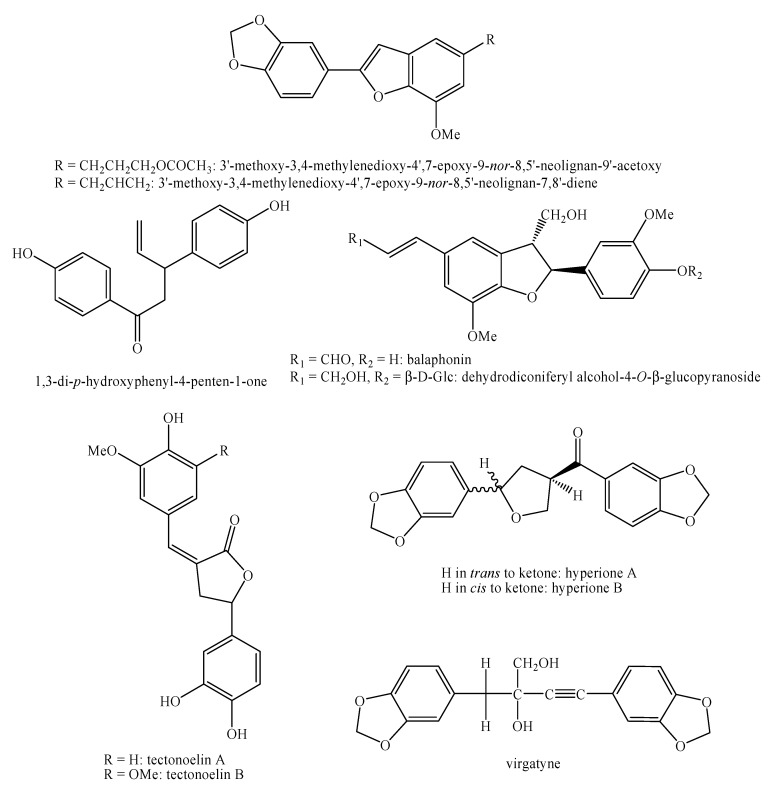
Isolated *nor*-lignans in the plant kingdom—part 4.

Pilosidine, nyasicoside and curculigine (Figure 4), in low doses ranging from 1 to 30 mM, are able to induce a reversible facilitating effect on adrenaline evoked contractions [47]. Moreover, they all have a dose dependent vasoconstricting effect on rabbit aorta strips [48]. Their mechanism of action involves an interaction with the peripheral adrenergic system, in particular with α1 and β1 adrenoceptors [48].

(2*S*)-1-*O*-butyl-nyasicoside and nyasicoside (Figure 4) possess high effects against the ouabain-induced arrhythmia in the heart preparations of guinea pig at the doses of 3 μM, especially at the left atrium level.

(2*S*)-1-*O*-butyl-nyasicoside (Figure 4) has the same effect but in minor extent [41].

Lastly, 2-(2′-hydroxy-4′,6′-dimethoxyphenyl)-5-[(*E*)-propenyl]benzofuran (Figure 22) inhibits the vasodilatory effect produced by acetylcholine with an IC_50_ value equal to 31.2 μM. This effect is concentration-dependent. Moreover, the compound inhibits basal nitric oxide production [118].

### 4.12. Antistress

Negundin A (Figure 20) shows a very good effect in mice by greatly decreasing the number of writhes at the dose of 25 mg/kg. Moreover, it is able to reduce the blood glucose level and serum cholesterol level but at higher doses (50 and 100 mg/kg) [119].

### 4.13. Anti-Inflammatory

Egonol, homoegonol, homoegonol gentiobioside, homoegonol glucoside and egonol gentiobioside (Figure 3) were found to exert weak or medium effects against COX-1 and COX-2 with percentages of inhibition ranging from 1.3 for homoegonol gentiobioside against COX-1 to 35.7 of homoegonol glucoside against COX-1 at the concentration of 30 mM [110]. Yet, egonol (Figure 7) is able to reduce the mRNA expression levels of inducible nitric oxide synthase (iNOS), COX-2, interleukin-1β (IL-1β) and interleukin-6 (IL-6). The same effect was observed also for homoegonol (Figure 7) but in a minor extent [111].

Lastly, nyasol and 5-((*S*,*Z*)-1-(4-hydroxyphenyl)penta-1,4-dien-3-yl)-2,3-dimethoxyphenol (Figure 7) show high effects. In particular, nyasol is able to inhibit microsomal cells by 100% as well as COX-1 while it inhibits COX-2 by 19%. Conversely, 5-((*S*,*Z*)-1-(4-hydroxyphenyl)penta-1,4-dien-3-yl)-2,3-dimethoxyphenol (Figure 7) inhibits microsomal cells by 72% and COX-2 by 23% [21].

### 4.14. Cytotoxic

3′-methoxy-nyasin and nyasol (Figure 7) possess moderate effects against HO-8910 (human ovarian carcinoma) and Bel-7402 (human hepatoma) cell lines. In particular, the former compound shows IC_50_ values equal to 84.0 and 26.2 μM, respectively whereas the latter compound shows IC_50_ values equal to 30.6 and 29.4 μM, respectively [18]. Nyasol and 4′-*O*-methyl-nyasol (Figure 7) exert moderate effects against the rat glioma C-6 cell line with IC_50_ values equal to 19.02 and 20.21 mg/mL, respectively [81]. Nyasol (Figure 7) is also able to inhibit the basic fibroblast growth factor (bFGF) and the vascular endothelial growth factor (VEGF)-induced endothelial cell proliferation [11]. The mechanism of action is related to its strong estrogen receptor binding ability [11]. In addition, nyasol (Figure 7) has medium effects against the human HL60 cancer cell line with IC_50_ value equal to 15.5 μM [27]. Moreover, nyasol, 4′-*O*-methyl-nyasol and 3′′-methoxy-nyasol (Figure 7) have a modest effect on the inhibition of β-hexosaminidase release in RBL-2H3 cells stimulated by DNP-BSA with IC_50_ values ranging from 18.08 μM for the latter to 52.67 μM for the second compound. These values are higher than the control compound ketotifen, which owns an IC_50_ value equal to 10.12 μM. Conversely, 3′′-hydroxy-4′′-methoxy-4′′-dehydroxy-nyasol (Figure 7) is more efficient than the control displaying an IC_50_ value equal to 2.85 μM [12].

Egonol and homoegonol (Figure 3) exhibit medium effects against B16F10 (murine melanoma), MCF-7 (human breast adenocarcinoma), HepG2 (human hepatocellular liver carcinoma), HeLa (human cervical adenocarcinoma) and MO59J (human glioblastoma) cell lines. These effects were observed to be higher with the passing of time reaching their peaks after 72 h. Anyway, they were not better than the controls doxorubicin, camptotechin and etoposide [105]. For what concerns egonol (Figure 3), the results for MCF-7 and HeLa were confirmed in another study and it was also observed that it is active against theHL-60 (human leukemia) cell line with an IC_50_ value equal to 47.8 μM [108].

Agatharesinol acetonide (Figure 1) exhibits strong effects on the A549 cell line (non-small-cell lung cancer) with an IC_50_ value equal to 27.1 μM, quite higher than taxol 33.72 μM [35].

Sequirin C (Figure 7) exerts good effects against the HL-60 cell line with an IC_50_ value of 5.5 μM, which is comparable to that of cisplatin (2.0 μM) [33].

**Figure 6:**
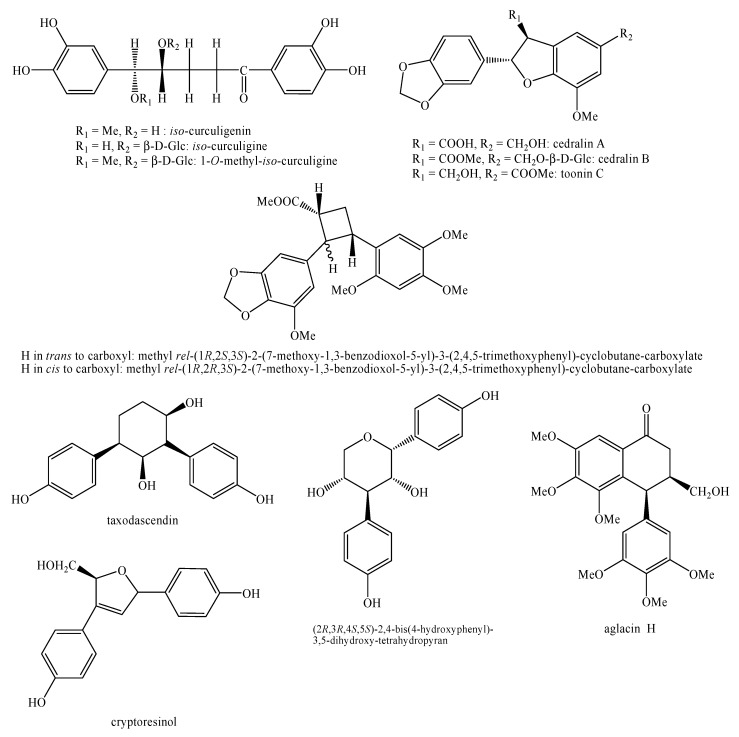
Isolated *nor*-lignans in the plant kingdom—part 5.

**Figure 7:**
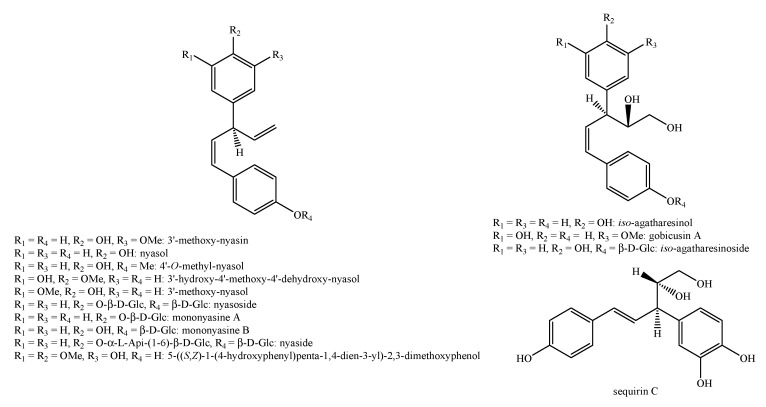
Isolated *nor*-lignans in the plant kingdom—part 6.

**Figure 8:**
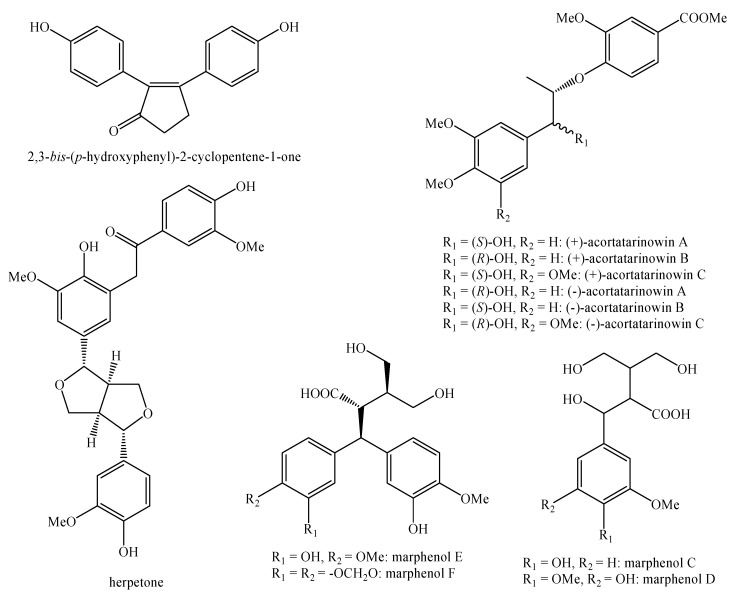
Isolated *nor*-lignans in the plant kingdom—part 7.

Cedralin A (Figure 6) has weak activities against the HL-60 and K562 (myelogenous leukemia) cell lines with IC_50_ values equal to 26.2 and 22.4 mg/mL, respectively [86].

Methyl *rel*-(1*R*,2*S*,3*S*)-2-(7-methoxy-1,3-benzodioxol-5-yl)-3-(2,4,5-trimethoxyphenyl)-cyclobutane -carboxylate and methyl *rel*-(1*R*,2*R*,3*S*)-2-(7-methoxy-1,3-benzodioxol-5-yl)-3- (2,4,5-trimethoxyphenyl)-cyclobutane-carboxylate (Figure 6) exert modest effects against the HepG2, A549 and HeLa cell lines with IC_50_ values equal to 38.0, 56.4 and 64.9 μM for the former in corresponding order, and 42.4, 66.3 and 77.7 μM for the latter in corresponding order [52].

Noralashinol B (Figure 15) exhibits a weak activity against the HepG2 cancer cell line with an IC_50_ value equal to 31.7 μM, which is higher than the positive control, methotrexate showing an IC_50_ value equal to 15.8 μM [90]. Its mechanism of action is via apoptosis [90].

**Figure 9:**
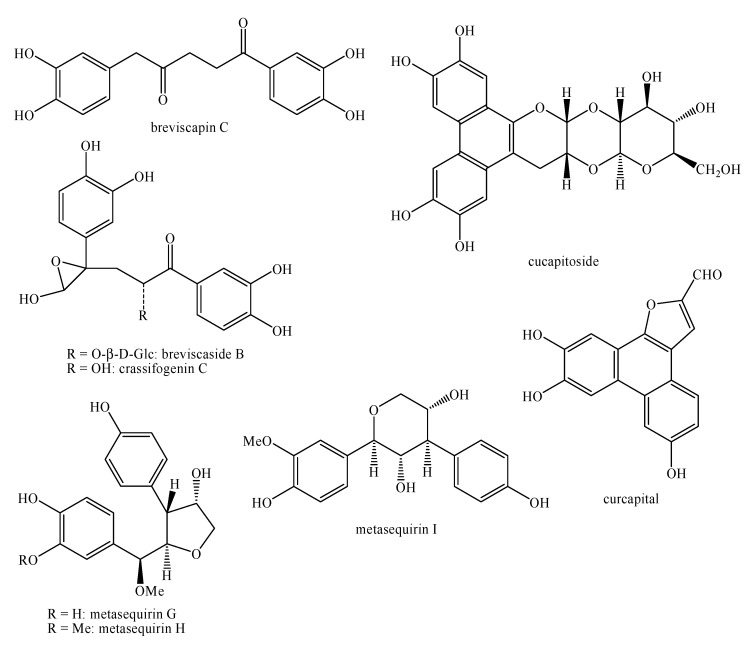
Isolated *nor*-lignans in the plant kingdom—part 8.

**Figure 10:**
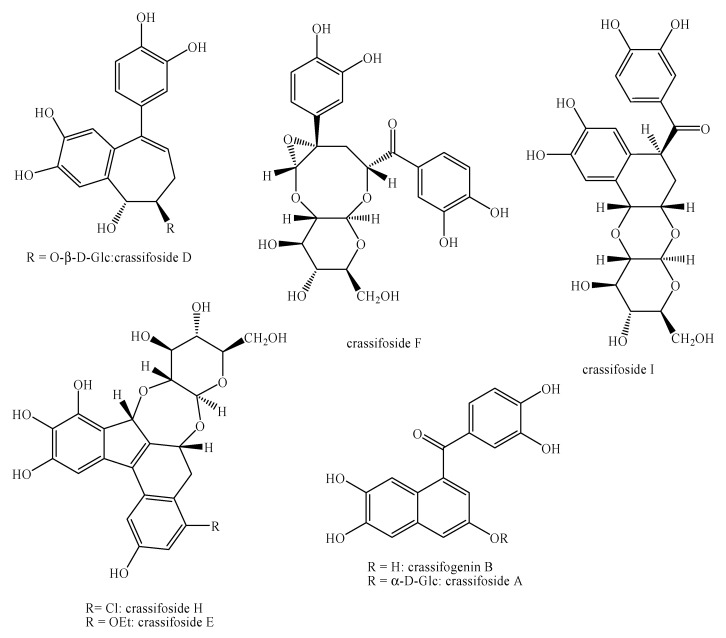
Isolated *nor*-lignans in the plant kingdom—part 9.

**Figure 11:**
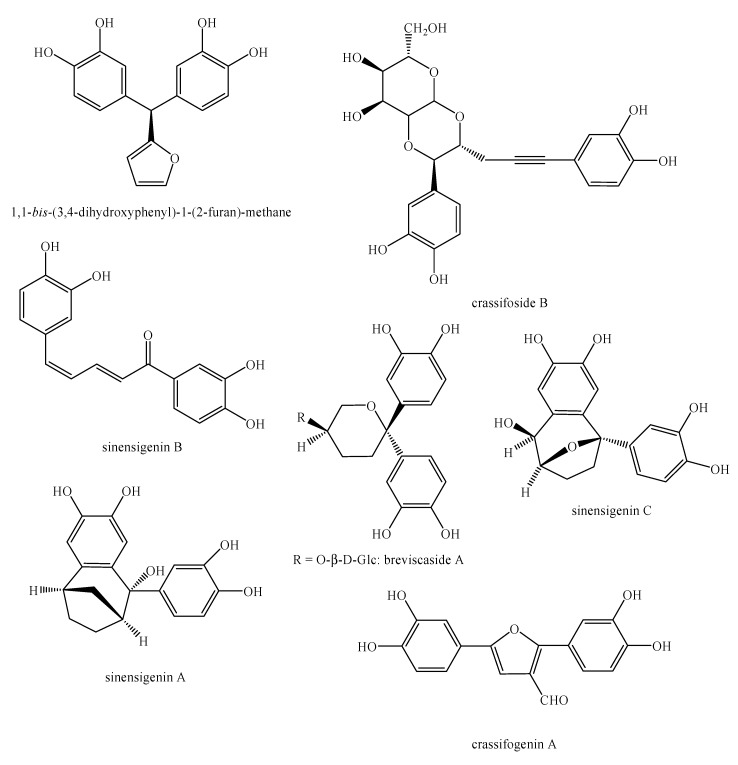
Isolated *nor*-lignans in the plant kingdom—part 10.

**Figure 12:**
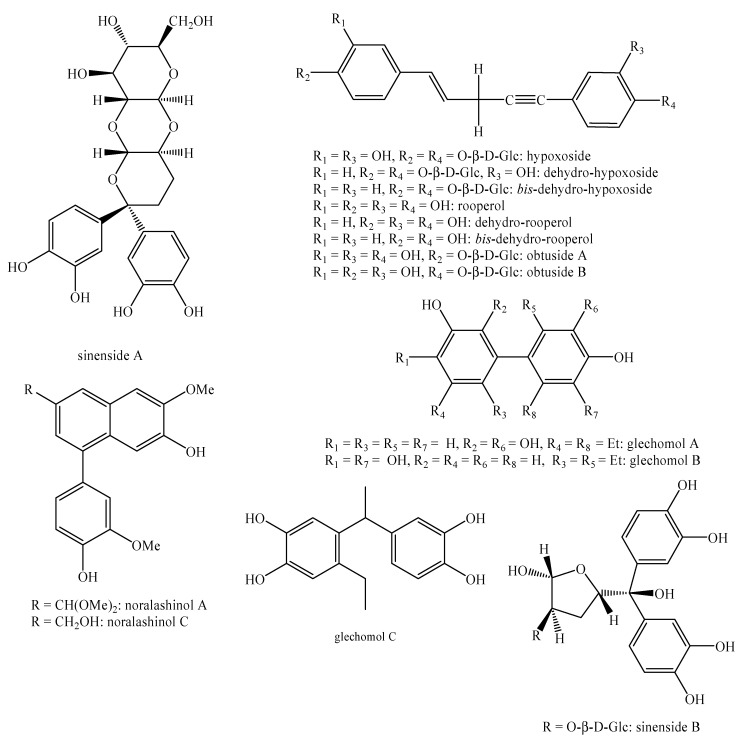
Isolated *nor*-lignans in the plant kingdom—part 11.

Metasequirin G, metasequirin H and metasequirin I (Figure 9) possess low cytotoxic effects against the A549 cell line with IC_50_ values close to 100 μM [34].

Chamaecypanone C (Figure 15) exerts potent effects against KB (human oral epidermoid carcinoma), HONE-1 (human nasopharyngeal carcinoma) and TSGH (human gastric carcinoma) cell lines with IC_50_ values equal to 0.19,0.24 and 0.52 μM, respectively [28].

Acorusin B (Figure 15) exert moderate effects against the CI-H1650 (non-small cell lung carcinoma), HepG2, BGC 823 (human stomach carcinoma), HCT-116 (human colon carcinoma) and MCF-7 cancer cell lines with IC_50_ values equal to 6.51, 4.80, 7.23, 8.81, 3.58 and 0.52 μM, respectively [6].

Yateresinol (Figure 17) is a decent cytotoxic compound against the human HL60 and Hepa G2 cancer cell lines with IC_50_ values higher than 20 μM [27].

Vitedoin A (Figure 20) exerts moderate effects against HCT116 cell lines with an IC_50_ value equal to 10.18 μM [74].

6-hydroxy-4-(4-hydroxy-3-methoxyphenyl)-3-hydroxymethyl-7-methoxy-3,4-dihydro-2-naphthaldehyde (Figure 20) shows high effects against HepG2 cell lines with an IC_50_ value equal to 8.24 μM, which is comparable to doxorubicin (IC_50_ = 6.49 μM) [74].

### 4.15. Phytotoxic

9′-*nor*-3′,4,4′-trihydroxy-3,5-dimethoxylign-7-eno-9,7′-lactone (Figure 18) is a phytotoxic compound against *Lactuca sativa* L. (lettuce) and *Lycopersicon esculentum* Mill. (tomato) preventing their development [101]. Moreover, only in the case of *L. esculentum*, it inhibits the shoot length [101].

### 4.16. Inhibition on Enzymes, Proteins and Platelet Aggregation

Negundin A, negundin B, 6-hydroxy-4-(4-hydroxy-3-methoxy)-3-hydroxymethyl-7-methoxy-3,4-dihydro-2-naphthaldehyde, vitrofolal E (Figure 20) and (+)-lyoniresinol (Figure 15) showed medium effects against tyrosinase with IC_50_ values equal to 10.06, 6.72, 7.81, 9.76 and 3.21 μM which are, anyway, higher than kojic acid (IC_50_ = 16.67μM) [71].

Indeed, negundin B (Figure 20) has potent effect against lipoxygenase with an IC_50_ value equal to 6.25 μM [70].

Vitrofolal E (Figure 20) shows also moderate effects against butyryl-cholinesterase with an IC_50_ value equal to 35.0 μM [70].

6-hydroxy-4-(4-hydroxy-3-methoxy)-3-hydroxymethyl-7-methoxy-3,4-dihydro-2-naphthaldehyde and vitrofolal E (Figure 20) have modest α-chymotrypsin (serine protease) competitive inhibitory effects with K_i_ values equal to 31.75 and 47.11 μM, respectively [72].

**Figure 14:**
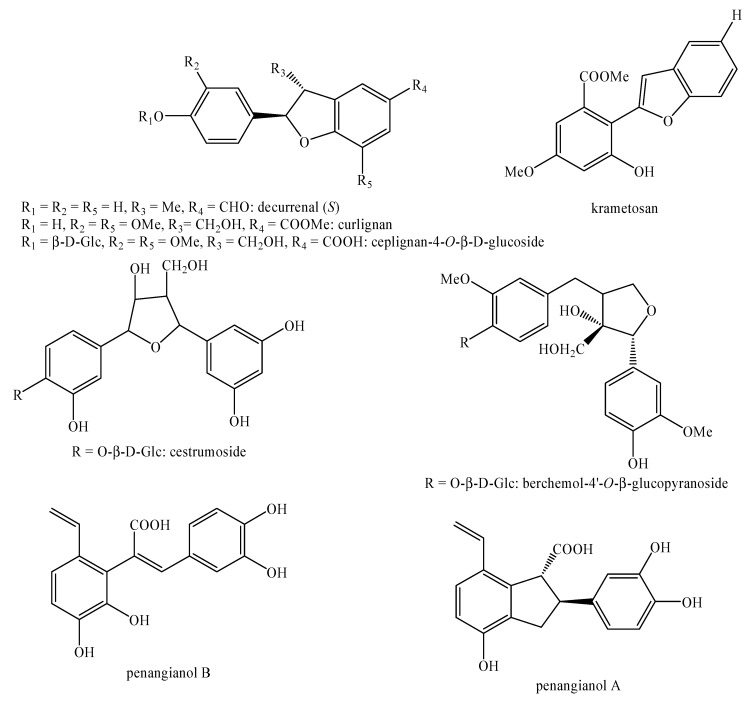
Isolated *nor*-lignans in the plant kingdom—part 13.

**Figure 15:**
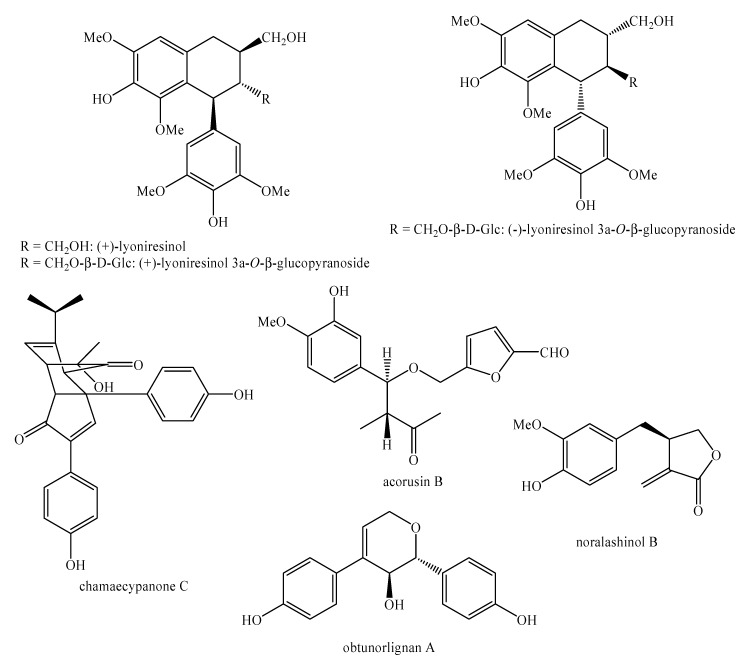
Isolated *nor*-lignans in the plant kingdom—part 14.

**Figure 16:**
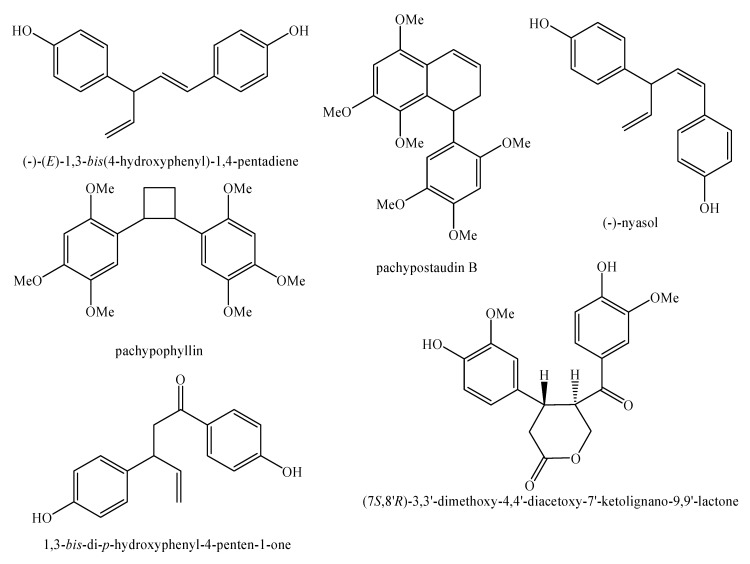
Isolated *nor*-lignans in the plant kingdom—part 15.

**Figure 17:**
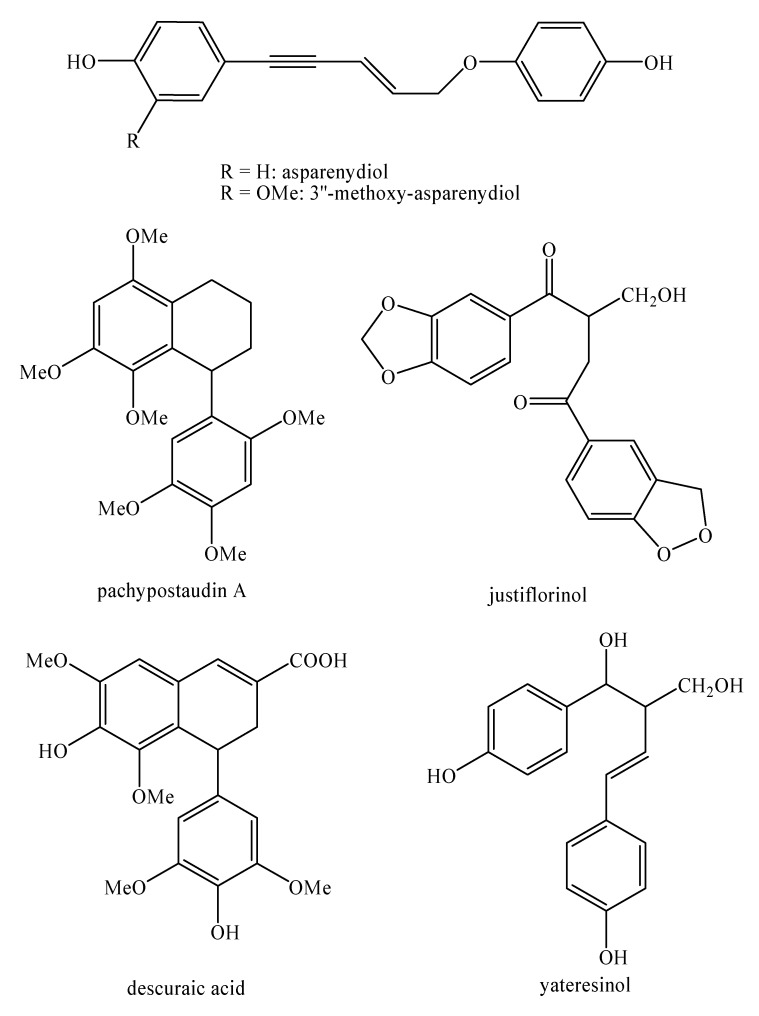
Isolated *nor*-lignans in the plant kingdom—part 16.

Cestrumoside (Figure 14) is a strong protein kinase C inhibitor in an animal food additive [120].

**Figure 18:**
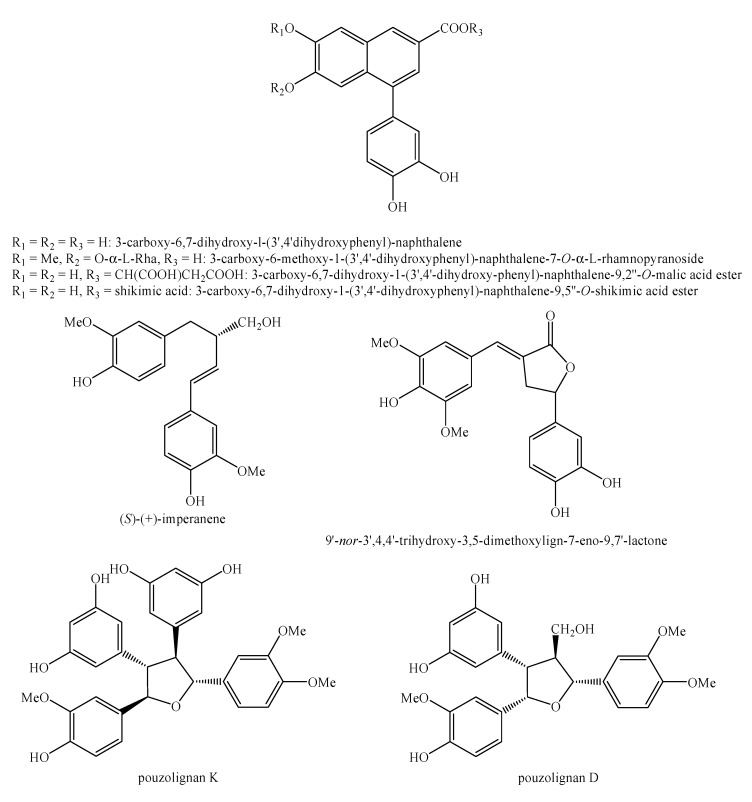
Isolated *nor*-lignans in the plant kingdom—part 17.

**Figure 19:**
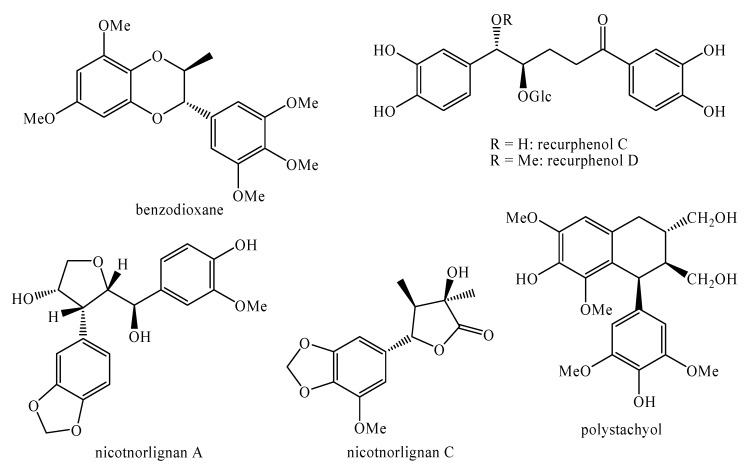
Isolated *nor*-lignans in the plant kingdom—part 18.

**Figure 20:**
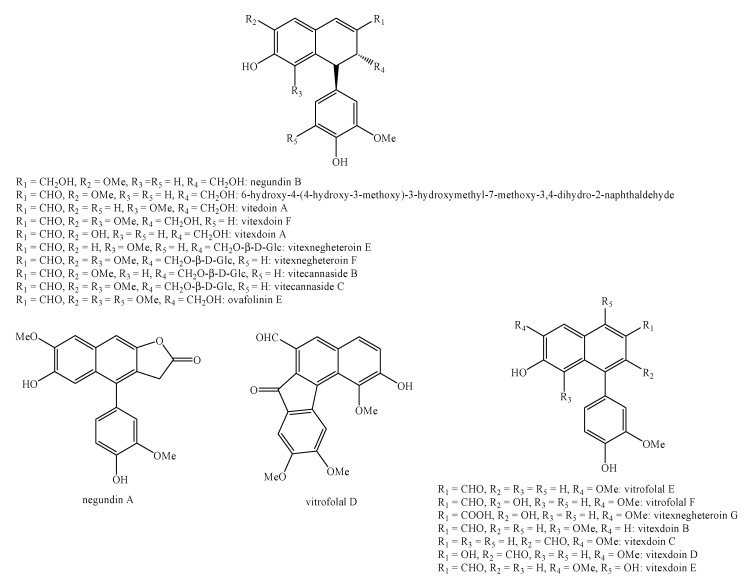
Isolated *nor*-lignans in the plant kingdom—part 19.

**Figure 21:**
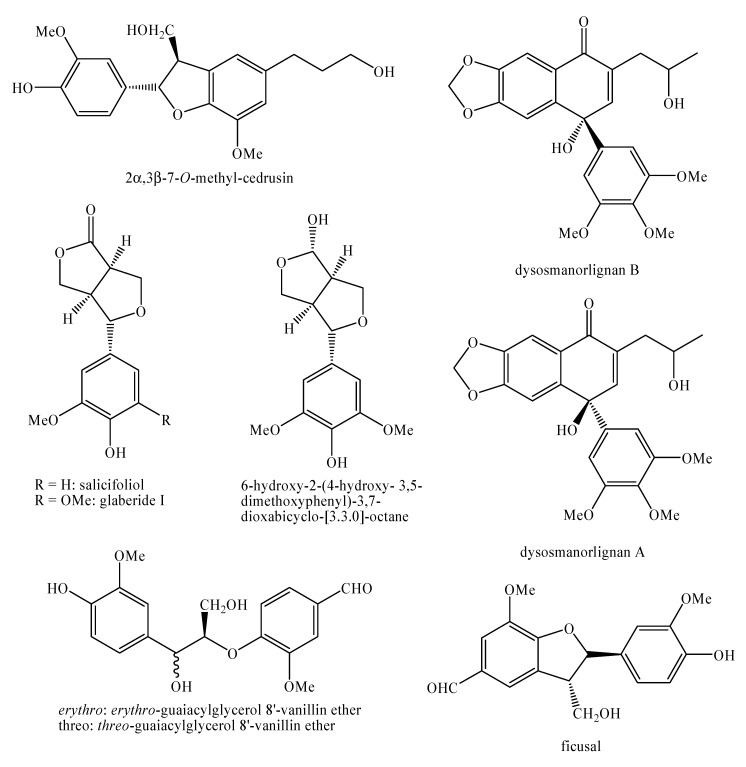
Isolated *nor*-lignans in the plant kingdom—part 20.

**Figure 22:**
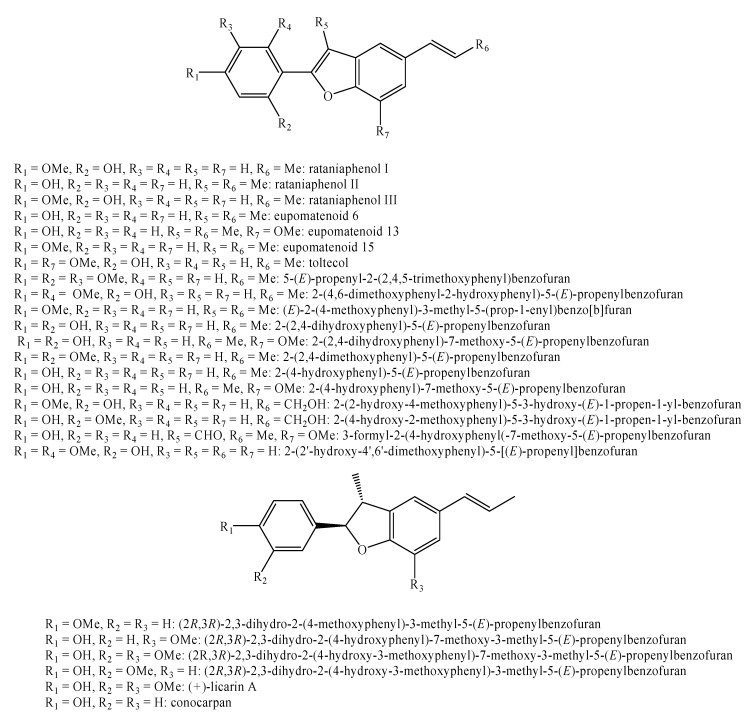
Isolated *nor*-lignans in the plant kingdom—part 21.

**Figure 23:**
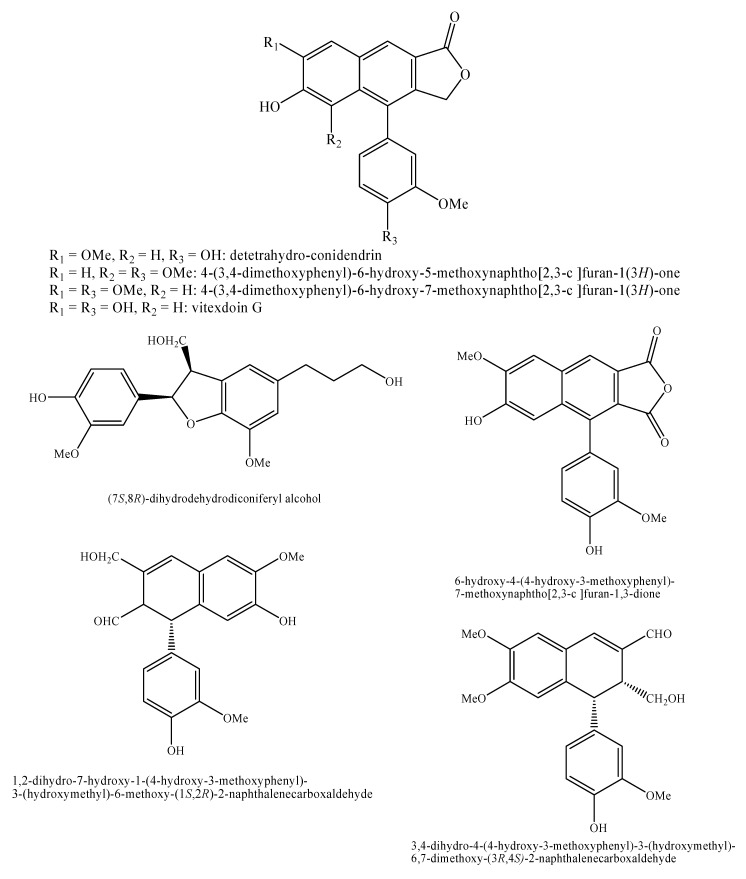
Isolated *nor*-lignans in the plant kingdom—part 22.

Lastly, (*S*)-(+)-imperanene (Figure 18) strongly inhibits tyronase isolated from HMV-II cells with an IC_50_ value equal to 1.85 mM [121]. Its mechanism of action is essentially identical to that of arbutin [121]. Moreover it shows a high effect in rabbits giving a complete inhibition at the concentration of 6 × 10^−4^ M when the platelet aggregation is induced by thrombin [96].

## 5. Conclusions

*Nor*-lignans have proven to be quite present in the plant kingdom. Nevertheless, some of them can be even considered to be chemotaxonomic markers. In addition, they are endowed with a vast number of biological activities with a myriad of possible application in several medicinal and pharmacological fields. Yet, not all the *nor*-lignans have been studied and discovered at the present. This review article means to be a first step towards the understanding of how important *nor*-lignans are as well as to be an incentive to continue their research and study.

## Figures and Tables

**Figure 13:**
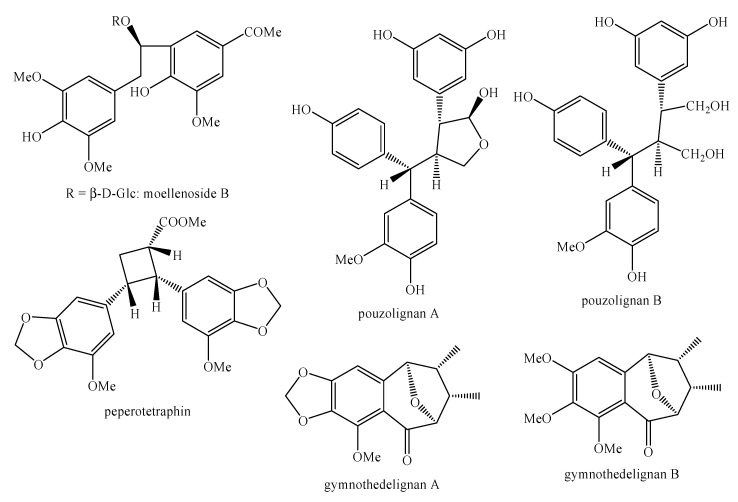
Isolated *nor*-lignans in the plant kingdom—part 12.

**Figure 24:**
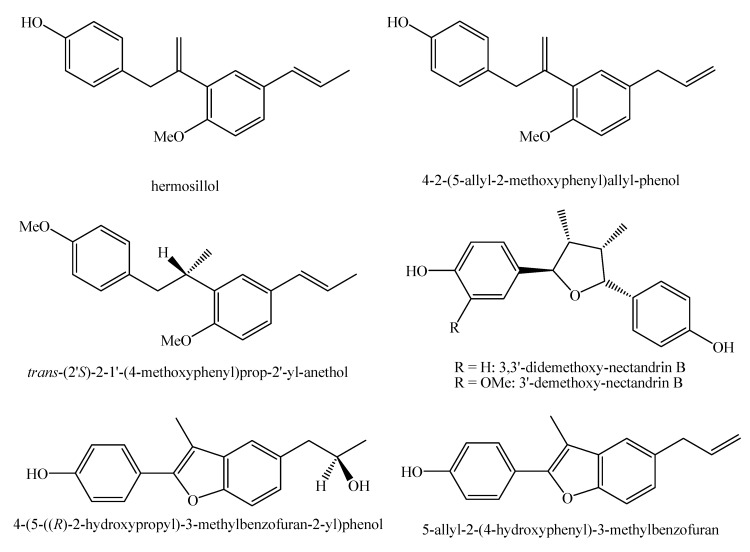
Isolated *nor*-lignans in the plant kingdom—part 23.

**Table 1:** of *nor*-lignans in the plant kingdom.

Family	Species	Studied Organs	Compounds	Methods	References
Acanthaceae	*Justicia patentiflora* Hemsl.	Leaves and stems	justiflorinol	SE, CC, HPLC, [α]_D_, UV, IR, NMR, MS	[4]
Acoraceae	*Acorus tatarinowii* Schott	Rhizomes	(*+*)-acortatarinowin A, (*+*)-acortatarinowin B, (*+*)-acortatarinowin C, (−)-acortatarinowin A, (−)-acortatarinowin B, (−)-acortatarinowin C	SE, CC, HPLC, ECD, [α]_D_, UV, IR, NMR, MS	[5]
			acorusin B	SE, CC, [α]_D_, UV, IR, NMR, MS	[6]
Annonaceae	*Duguetia confinis* (Engl. and Diels) Chatrou	Bark	pachypostaudin A, pachypostaudin B	SE, CC, TLC, NMR, MS	[7]
	*Pachypodanthium staudlii* (Engl. and Diels) Engl. and Diels	Bark	pachypostaudin A, pachypostaudin B, pachypophyllin	SE, MP, NMR, MS	[8]
Araucariaceae	*Araucaria angustifolia* (Bertol.) Kuntze	Knot resin	2,3-*bis*-(*p*-hydroxyphenyl)-2-cyclopentene-1-one, nyasol, cryptoresinol	SE, CC, HPLC, TLC, UV, IR, NMR, MS	[9]
Asparagaceae	*Anemarrhena asphodeloides* Bunge	Rhizomes	nyasol, 4′-*O*-methyl-nyasol, 1,3-di-*p*-hydroxyphenyl-4-penten-1-one	SE, CC, LC, [α]_D_, NMR, MS	[10,11]
			nyasol, 4′-*O*-methyl-nyasol, 3″-methoxy-nyasol, 3″-hydroxy-4″-methoxy-4″-dehydroxy-nyasol	SE, CC, NMR, MS	[12]
			nyasol	SE, CC, HPLC, UV, NMR, MS	[13]
	*Asparagus africanus* Lam.	Roots	nyasol	CC, LC, HPLC, NMR, MS	[14]
	*Asparagus cochinchinensis* (Lour.) Merr.	Roots	*iso*-agatharesinoside, *iso*-agatharesinol	SE, CC, [α]_D_, UV, IR, NMR, MS	[15]
		Tubers	nyasol	SE, CC, [α]_D_, IR, NMR, MS	[16]
		Roots	3′-hydroxy-4′-methoxy-4′-dehydroxy-nyasol, nyasol, 3′-methoxy-nyasol, 1,3-*bis*-di-*p*-hydroxyphenyl-4-penten-1-one, asparenydiol, 3′′-methoxy-asparenydiol	SE, CC, [α]_D_, UV, IR, NMR, MS	[17]
	*Asparagus gobicus* N.A.Ivanova ex Grubov	Roots	3′-methoxy-nyasin, *iso*-agatharesinol, gobicusin A, gobicusin B, nyasol, 4-[5-(4-methoxyphenoxy)-3-penten-1-ynyl]phenol, sequirinC	SE, CC, [α]_D_, UV, IR, NMR, MS	[18]
	*Asparagus racemosus* Willd.	Whole plant	*iso*-agatharesinol, gobicusin A	SE, CC, NMR, MS	[19]
	*Drimiopsis burkei* Baker	Bulbs	(−)-nyasol	SE, CC, [α]_D_, NMR	[20,21]
	*Drimiopsis maculata* Lindl. and Paxton	Bulbs	(−)*-(E)-*1,3-*bis*(4-hydroxyphenyl)-1,4-pentadiene	SE, CC, [α]_D_, NMR	[20]
	*Ledebouria ovatifolia* (Baker) Jessop	Whole plant	5-((*S*,*Z*)-1-(4-hydroxyphenyl)penta-1,4-dien-3-yl)-2,3-dimethoxyphenol	n.r.	[21]
	*Rhodocodon campanulatus* H. Perrier	Bulbs	(7*S*,8′*R*)-3,3′-dimethoxy-4,4′-diacetoxy-7′-ketolignano-9,9′-lactone	SE, CC, [α]_D_, ECD, IR, NMR, MS	[22]
Berberidaceae	*Dysosma versipellis* (Hance) M.Cheng	Roots	dysosmanorlignan A, dysosmanorlignan B	SE, CC, HPLC, TLC, [α]_D_, IR, UV, NMR, MS	[23]
Brassicaceae	*Descurainia sophia* (L.) Webb ex Prantl	Roots	descuraic acid	SE, LC, CC, [α]_D_, NMR, MS	[24]
Compositae	*Saussurea macrota* Franch	Whole plant	egonol	SE, CC, TLC, [α]_D_, IR, NMR, MS	[25]
Cucurbitaceae	*Herpetospermum pedunculosum* (Ser.) C.B. Clarke	Whole plant	herpetone	SE, CC, HPLC, IR, UV, NMR, MS	[26]
Cupressaceae	*Chamaecyparis formosensis* Matsum.	Wood	yateresinol, nyasol	SE, CC, UV, IR, NMR	[27]
	*Chamaecyparis obtusa* var. *formosana* (Hayata) Hayata	Heartwood	chamaecypanone C, obtunorlignan A	SE, CC, [α]_D_, UV, IR, NMR, MS	[28]
		Wood	*trans*-nyasol	SE, [α]_D_, MP, IR, MS	[29]
	*Cryptomeria japonica* (Thunb. ex L.f.) D.Don	Whole plant	agatharesinol	IM	[30]
		n.a.	yateresinol	n.a.	[31]
	*Libocedrus yateensis* Guillaumin	Heartwood	yateresinol, nyasol	SE, CC, [α]_D_, IR, NMR, MS	[32]
	*Metasequoia glyptostroboides* Huand W.C.Cheng	Stems and leaves	metasequirin D, metasequirin E, metasequirin F, sequosempervirin B, sequosempervirin D, sequosempervirin F, agatharesinol, agatharesinol acetonide, sequirin C, nyasol	SE, CC, LC, HPLC, [α]_D_, IR, NMR, MS	[33]
		Branches and stems	metasequirin G, metasequirin H, metasequirin I	SE, CC, LC, HPLC, [α]_D_, UV, IR, NMR, MS	[34]
	*Sequoia sempervirens* (D.Don) Endl.	Branches and leaves	sequosempervirin B, sequosempervirin C, sequosempervirin D, sequosempervirin E, sequosempervirin F, sequosempervirin G, agatharesinol, agatharesinol acetonide, sugiresinol	SE, CC, [α]_D_, UV, NMR, MS	[35]
		Heartwood	sugiresinol, sequirin B, sequirin C, sequirin D, dimethyl-agatharesinol	SE, CC, LC, NMR, MS	[36]
	*Sequoiadendron giganteum* (Lindl.) J.Buchholz	Heartwood	sequirin E, sequirin F, sequirin G, agatharesinol, dimethyl-agatharesinol, dimethyl-agatharesinol acetonide	SE, CC, TLC, NMR, MS	[36]
	*Taxodium ascendens* Brongn.	Leaves and branches	(2*R*,3*R*,4*S*,5*S*)-2,4-bis(4-hydroxyphenyl)-3,5-dihydroxy-tetrahydropyran, sequosemperverin B, agatharesinol, cryptoresinol	SE, CC, [α]_D_, IR, UV, NMR, MS	[37]
	*Taxodium distichum* var. *imbricatum* (Nutt.) Croom	Leaves and branches	taxodascendin, cryptoresinol, sequosempervirin B, agatharesinol	SE, CC, NMR, IR, UV, MS	[38]
Hypericaceae	*Hypericum chinense* L.	Leaves	hyperione A, hyperione B	SE, CC, [α]_D_,IR, NMR, MS	[39]
Hypoxidaceae	*Curculigo breviscapa* S.C.Chen	Rhizomes	breviscapin C, breviscaside B, curcapital, capituloside, pilosidine, cucapitoside, crassifoside H, crassifoside F	SE, CC, LC, [α]_D_, IR, UV, NMR, MS	[40]
	*Curculigo capitulata* (Lour.) Kuntze	Rhizomes	(2*S*)-1-*O*-butyl-*iso*-nyasicoside, (2*S*)-1-*O*-butyl-nyasicoside, nyasicoside, 3′′-dehydroxy-nyasicoside, 1-*O*-methyl-nyasicoside, curlignan	SE, CC, IR, UV, CD, NMR, MS	[41]
			capituloside, curculigenin, *iso*-curculigenin, curculigine, *iso*-curculigine, 1-*O*-methyl-curculigine, 1-*O*-methyl-*iso*-curculigine	SE, TLC, CC, IR, UV, NMR, MS	[42]
			crassifoside I, sinensigenin C, 1,1-*bis*-(3,4-dihydroxyphenyl)-1-(2-furan)-methane, crassifogenin B, crassifoside A, breviscaside A, crassifoside D, curcapital	SE, CC, [α]_D_, IR, UV, NMR, MS	[43]
	*Curculigo crassifolia* (Baker) Hook.f.	Rhizomes	crassifogenin C, curcapital, crassifoside E, crassifoside F	SE, CC, IR, UV, NMR, MS	[44]
			1-*O*-methyl-nyasicoside, 1-*O*-methyl-*iso*-nyasicoside, (1*R*)-crassifogenin D, (1*S*)-crassifogenin D	SE, CC, LC, [α]_D_, IR, UV, NMR, MS	[45]
			crassifogenin A, crassifogenin B, crassifoside A, crassifoside B	SE, CC, [α]_D_, IR, UV, NMR, MS	[46]
	*Curculigo pilosa* (Schumach. and Thonn.) Engl.	Rhizomes	nyasicoside, curculigine, pilosidine	SE, CC, [α]_D_, IR, UV, NMR, MS	[47,48]
	*Curculigo recurvata* W.T.Aiton	Rhizomes	curculigine, *iso*-curculigine, 1-*O*-methyl-curculigine, 1-*O*-methyl-*iso*-curculigine, nyasicoside	SE, CC, CE, CD, NMR, MS,	[49,50]
	*Curculigo sinensis* S.C.Chen	Rhizomes	sinensigenin A, sinensigenin B, crassifogenin B, cucapitoside, crassifoside B, crassifoside H, curculigine, *iso*-curculigine	SE, CC, LC, [α]_D_, IR, UV, NMR, MS	[51]
			sinenside A, sinenside B, crassifoside D, capituloside, 1-*O*-methyl-nyasicoside, 1-*O*-methyl-*iso*-nyasicoside, 1-*O*-methyl-curculigine, 1-*O*-methyl-*iso*-curculigine	SE, CC, [α]_D_, IR, UV, NMR, MS	[52]
	*Hypoxis angustifolia* Lam.	Rhizomes	nyasol, hypoxoside, nyasosidenyaside, mononyasine A, mononyasine B	SE, CC, [α]_D_, IR, UV, NMR, MS	[53]
	*Hypoxis hemerocallidea* Fisch., C.A.Mey. and Avé-Lall.	Rhizomes	hypoxoside, dehydroxy-hypoxoside, *bis*-dehydroxy-hypoxoside, rooperol, dehydroxy-rooperol, *bis*-dehydroxy-rooperol	SE, HPLC, LC, UV, NMR, MS	[54]
	*Hypoxis interjecta* Nel	Rhizomes	interjectin	SE, CC, [α]_D_, IR, UV, NMR, MS	[55]
	*Hypoxis multiceps* Buchinger ex Baker	Rhizomes	interjectin	SE, CC, [α]_D_, IR, UV, NMR, MS	[55]
	*Hypoxis nyasica* Baker	Rhizomes	nyasicoside, mononyasine A, mononyasine B, nyaside, hypoxoside, nyasoside	SE, CC, [α]_D_, IR, UV, NMR, MS	[56,57,58]
	*Hypoxis obtusa* Burch. ex Ker Gawl.	Rhizomes	hypoxoside	SE, CC, NMR, MS	[59]
			rooperol, obtuside A, obtuside B	SE, CC, [α]_D_, IR, UV, NMR, MS	[60]
Jungermanniaceae	*Jungermannia exsertifolia* Stephani	Whole plant	3-carboxy-6,7-dihydroxy-l-(3′,4′dihydroxyphenyl)-naphthalene, 3-carboxy-6,7-dihydroxy-1-(3′,4′-dihydroxyphenyl)-naphthalene-9,5′′-*O*-shikimic acid ester	SE, CC, LC, HPLC, [α]_D_, NMR, MS	[61]
Krameriaceae	*Krameria cytisoides* Cav.	Roots	(2*R*,*3R*)-2,3-dihydro-2-(4-hydroxy-3-methoxyphenyl)-3-methyl-5-(*E*)-propenylbenzofuran, (2*R*,3*R*)-2,3-dihydro-2-(4-hydroxy-3-methoxyphenyl)-7-methoxy-3-methyl-5-(E)-propenylbenzofuran, (2*R*,3*R*)-2,3-dihydro-2-(4-hydroxyphenyl)-7-methoxy-3-methyl-5-(*E*)-propenylbenzofuran, conocarpan, rataniaphenol II, eupomatenoid 13, 3-formyl-2-(4-hydroxyphenyl(-7-methoxy-5-(*E*)-propenylbenzofuran, 2-(2,4-dimethoxyphenyl)-5-(*E*)-propenylbenzofuran, rataniaphenol I, toltecol,2-(4-hydroxyphenyl)-7-methoxy-5-(*E*)-propenylbenzofuran, 2-(2,4-dihydroxyphenyl)-5-(*E*)-propenylbenzofuran, 2-(2,4-dihydroxyphenyl)-7-methoxy-5-(*E*)-propenylbenzofuran, olmecol,3,3′-didemethoxy-nectandrin B, 3′-demethoxy-nectandrin B	SE, CC, TLC, UV, IR, NMR, MS	[62]
	*Krameria grayi* Rose and Painter	Roots	rataniaphenol I, eupomatenoid 6, 2-(2,4-dihydroxyphenyl)-5-(*E*)-propenylbenzofuran, (*E*)-2-(4-methoxyphenyl)-3-methyl-5-(prop-1-enyl)benzo[b]furan, rataniaphenol III, 2-(2,4-dimethoxyphenyl)-5-(*E*)-propenylbenzofuran, 2-(4-hydroxyphenyl)-5-(*E*)-propenylbenzofuran, 2-(4-hydroxy-2-methoxyphenyl)-5-3-hydroxy-(*E*)-1-propen-1-yl-benzofuran, 2-(2-hydroxy-4-methoxyphenyl)-5-3-hydroxy-(*E*)-1-propen-1-yl-benzofuran, (2*R*,3*R*)-2,3-dihydro-2-(4-methoxyphenyl)-3-methyl-5-(*E*)-propenylbenzofuran, (2*R*,3*R*)-2,3-dihydro-2-(4-hydroxyphenyl)-7-methoxy-3-methyl-5-(*E*)-propenylbenzofuran, (*+*)-licarin A, (2*R*,3*R*)-2,3-dihydro-2-(4-hydroxy-3-methoxyphenyl)-3-methyl-5-(*E*)-propenylbenzofuran, 4-(5-((*R*)-2-hydroxypropyl)-3-methylbenzofuran-2-yl)phenol	SE, CC, TLC, IR, UV, NMR, MS	[63]
	*Krameria ixine* L.	Roots	conocarpan, ratanhiaphenol I, ratanhiaphenol II, 2-(4,6-dimethoxyphenyl-2-hydroxyphenyl)-5-(*E*)-propenylbenzofuran, 2-(4-hydroxyphenyl)-5-((*E*)-prop-2-en-1-yl)benzofuran, 2-(2,4-dihydroxyphenyl)-5-((*E*)-prop-2-en-1-yl)benzofuran, 5-(*E*)-propenyl-2-(2,4,5-trimethoxyphenyl)benzofuran, eupomatenoid15, 5-allyl-2-(4-hydroxyphenyl)-3-methylbenzofuran, hermosillol, 4-2-(5-allyl-2-methoxyphenyl)allyl-phenol, *trans*-(2′*S*)-2-1′-(4-methoxyphenyl)prop-2′-yl-anethol, 3,3′-didemethoxy-nectandrin B	SE, CC, TLC, [α]_D_, CD, UV, IR, NMR, MS	[64]
	*Krameria tomentosa* A. St.-Hil.	Roots	krametosan, ratanhiaphenol II,2-(2′-hydroxy-4′,6′-dimethoxyphenyl)-5-[(*E*)-propenyl]benzofuran, conocarpan, decurrenal (*S*)	SE, CC, [α]_D_, IR, NMR, MS	[65]
Lamiaceae	*Glechoma longituba* (Nakai) Kuprian.	Whole plant	glechomol A, glechomol B, glechomol C	SE, CC, [α]_D_, IR, UV, NMR, MS	[66]
	*Tectona grandis* L.f.	Leaves	balaphonin, tectonoelin A, tectonoelin B	SE, CC, HPLC, IR, NMR, MS	[67]
	*Vitex negundo* var. *cannabifolia* (Siebold and Zucc.) Hand.-Mazz.	Fruits	vitrofolal E, vitrofolal F	SE, CC, HPLC, NMR, MS	[68]
		Seeds	6-hydroxy-4-(4-hydroxy-3-methoxyphenyl)-3-hydroxymethyl-7-methoxy-3,4-dihydro-2-naphthaldehyde, vitexdoin A, vitexdoin E, vitexdoin C, vitexdoin D, vitexdoin B, vitexdoin F, vitrofolal A, vitrofolal B, vitrofolal E, vitrofolal F, negundin B, detetrahydro-conidendrin, vitedoin A, negundin B, 4-(3,4-dimethoxyphenyl)-6-hydroxy-5-methoxynaphtho[2,3-c ]furan-1(3*H*)-one, 4-(3,4-dimethoxyphenyl)-6-hydroxy-7-methoxynaphtho[2,3-c ]furan-1(3*H*)-one, 6-hydroxy-4-(4-hydroxy-3-methoxyphenyl)-7-methoxy-naphtho[2,3-c ]furan-1,3-dione, 1,2-dihydro-7-hydroxy-1-(4-hydroxy-3-methoxyphenyl)-3-(hydroxymethyl)-6-methoxy-(1*S*,2*R*)-2-naphthalenecarboxaldehyde,3,4-dihydro-4-(4-hydroxy-3-methoxyphenyl)-3-(hydroxymethyl)-6,7-dimethoxy-(3*R*,4*S*)-2-naphthalenecarboxaldehyde	SE, CC, LC, HPLC, [α]_D_, CD, NMR, MS	[69]
	*Vitex negundo* L.	Roots	negundin A, negundin B, 6-hydroxy-4-(4-hydroxy-3-methoxy)-3-hydroxymethyl-7-methoxy-3,4-dihydro-2-naphthaldehyde, (+)-lyoniresinol,(+)-lyoniresinol 3a-*O*-β-glucopyranoside, vitrofolal E, vitrofolal F	SE, CC, TLC, IR, UV, NMR, MS	[70,71]
			negundin A, negundin B, 6-hydroxy-4-(4-hydroxy-3-methoxy)-3-hydroxymethyl-7-methoxy-3,4-dihydro-2-naphthaldehyde, (+)-lyoniresinol,(+)-lyoniresinol 3a-*O*-β-glucopyranoside, vitrofolal E	SE, CC, [α]_D_, IR, UV, NMR, MS	[72]
		Seeds	vitedoin A, 6-hydroxy-4-(4-hydroxy-3-methoxyphenyl)-3-hydroxymethyl-7-methoxy-3,4-dihydro-2-naphthaldehyde, detetrahydro-conidendrin, vitrofolal E, vitrofolal F, 2α,3β-7-*O*-methyl-cedrusin	SE, CC, [α]_D_, NMR, MS	[73]
			vitexnegheteroin E, vitexnegheteroin F, vitexnegheteroin G, vitecannaside B, 6-hydroxy-4-(4-hydroxy-3-methoxyphenyl)-3-hydroxymethyl-7-methoxy-3,4-dihydro-2-naphthaldehyde, vitedoin A, vitexdoin A	SE, CC, [α]_D_, CD, UV, IR, NMR, MS	[74]
			vitexdoin A, vitexdoin B, vitexdoin C, vitexdoin D, vitexdoin E, 6-hydroxy-4-(4-hydroxy-3-methoxyphenyl)-3-hydroxymethyl-7-methoxy-3,4-dihydro-2-naphthaldehyde, vitrofolal E, vitrofolal F	SE, CC, LC, [α]_D_, CD, UV, IR, NMR, MS	[75]
		Aerial parts	vitedoin A, 6-hydroxy-4-(4-hydroxy-3-methoxyphenyl)-3-hydroxymethyl-7-methoxy-3,4-dihydro-2-naphthaldehyde, 2α,3β-7-*O*-methyl-cedrusin, vitexdoin F, vitexdoin A, (–)-lyoniresinol-3a-*O*-β-d-glucopyranoside, (+)-lyoniresinol-3*a*-*O*-*β*-d-glucopyranoside, vitecannaside B, ovafolinin E, (7*S*,8*R*)-dihydrodehydrodiconiferyl alcohol, vitecannaside C, vitexdoin G	SE, CC, LC, [α]_D_, UV, IR, NMR, MS	[76]
	*Vitex rotundifolia* L.f.	Roots	vitrofolal A, vitrofolal B, vitrofolal C, vitrofolal D, vitrofolal E, vitrofolal F, detetrahydro-conidendrin, 4-(3,4-dimethoxyphenyl)-6-hydroxy-5-methoxynaphtho[2,3-c ]furan-1(3*H*)-one, 4-(3,4-dimethoxyphenyl)-6-hydroxy-7-methoxynaphtho[2,3-c ]furan-1(3*H*)-one	SE, CC, MP, UV, IR, NMR, MS	[77]
Lauraceae	*Nectandra lineata* (Kunth) Rohwer	Young leaves	3′-methoxy-3,4-methylenedioxy-4′,7-epoxy-9-nor-8,5′-neolignan-9′-acetoxy, 3′-methoxy-3,4-methylenedioxy-4′,7-epoxy-9-nor-8,5′-neolignan-7,8′-diene	SE, CC, IR, NMR, MS	[78]
Lepidoziaceae	*Bazzania trilobata* (L.) Gray	Whole plant	3-carboxy-6,7-dihydroxy-l-(3′,4′dihydroxyphenyl)-naphthalene	SE, CC, HPLC, NMR, MS	[79]
	*Lepidozia incurvata* Lindenb.	Whole plant	3-carboxy-6,7-dihydroxy-l-(3′,4′dihydroxyphenyl)-naphthalene, 3-carboxy-6-methoxy-1-(3′,4′-dihydroxyphenyl)-naphthalene-7-*O*-α-L-rhamnopyranoside	SE, CC, LC, HPLC, [α]_D_, NMR, MS	[61]
	*Lepidozia reptans* (L.) Dumort.	n.a.	3-carboxy-6,7-dihydroxy-l-(3′,4′dihydroxyphenyl)-naphthalene	n.a.	[80]
Lophocoleaceae	*Chiloscyphus polyanthos* (L.) Corda	Whole plant	3-carboxy-6,7-dihydroxy-l-(3′,4′dihydroxyphenyl)-naphthalene, 3-carboxy-6,7-dihydroxy-1-(3′,4′-dihydroxy-phenyl)-naphthalene-9,2′′-*O*-malic acid ester	SE, CC, LC, HPLC, [α]_D_, NMR, MS	[80]
Lythraceae	*Sonneratia caseolaris* (L.) Engl.	Fruits	nyasol, 4′-*O*-methyl-nyasol	SE, CC, TLC, NMR, MS	[81]
	*Sonneratia ovata* Backer	Fruits	nyasol, 4′-*O*-methyl-nyasol	SE, CC, TLC, NMR, MS	[81]
	*Trapa natans* L.	Whole plant	nyasol	SE, CC, [α]_D_, IR, NMR, MS	[82]
Magnoliaceae	*Magnolia odora* (Chun) Figlar and Noot.	Twigs	glaberide I, salicifoliol, 6-hydroxy-2-(4-hydroxy-3,5-dimethoxyphenyl)-3,7-dioxabicyclo-[3.3.0]-octane, ficusal, *erythro*-guaiacylglycerol 8′-vanillin ether, *threo*-guaiacylglycerol 8′-vanillin ether	SE, CC, LC, HPLC, NMR, MS	[83]
Malvaceae	*Urena lobata* L.	Aerial parts	ceplignan-4-*O*-β-d-glucoside	SE, CC, [α]_D_, IR, UV, NMR, MS	[84]
Meliaceae	*Aglaia cordata* Hiern	Stem barks	aglacin H	SE, CC, HPLC, NMR, MS	[85]
	*Cedrela sinensis* Juss.	Leaves	cedralin A, cedralin B	SE, IR, UV, NMR, MS	[86]
	*Toona sinensis*(Juss.) M.Roem.	Roots	toonin C	SE, CC, HPLC, [α]_D_, IR, NMR, MS	[87]
Oleaceae	*Syringa pinnatifolia* Hemsl.	Stem barks	noralashinol A, vitrofolal E	SE, CC, UV, IR, NMR, MS	[88,89]
			noralashinol B, noralashinol C	SE, CC, LC, [α]_D_, UV, IR, ECD, NMR, MS	[90]
Pelliaceae	*Pellia epiphylla* (L.) Corda	Gametophytes	3-carboxy-6,7-dihydroxy-l-(3′,4′dihydroxyphenyl)-naphthalene	SE, CC, IR, NMR, MS	[91]
Phyllanthaceae	*Phyllanthus virgatus* G.Forst.	Whole plant	virgatyne	SE, CC, LC, [α]_D_, UV, NMR, MS	[92]
Piperaceae	*Peperomia tetraphylla* (G.Forst.) Hook. and Arn.	Whole plant	methyl *rel*-(1*R*,2*S*,3*S*)-2-(7-methoxy-1,3-benzodioxol-5-yl)-3-(2,4,5-trimethoxyphenyl)-cyclobutane-carboxylate, methyl *rel*-(1*R*,2*R*,3*S*)-2-(7-methoxy-1,3-benzodioxol-5-yl)-3-(2,4,5-trimethoxyphenyl)-cyclobutane-carboxylate	SE, CC, LC, [α]_D_, UV, IR, CD, NMR, MS	[93]
			peperotetraphin	SE, CC, LC, [α]_D_, UV, IR, NMR, MS	[94]
	*Piper obliquum* Ruiz and Pav.	Leaves	justiflorinol	SE, CC, [α]_D_, UV, IR, NMR, MS	[95]
Poaceae	*Imperata cilindrica* (L.) Raeusch.	Rhizomes	(*S*)-(+)-imperanene	SE, CC, [α]_D_, NMR, MS	[96]
Saururaceae	*Gymnotheca chinensis* Decne.	Whole plant	gymnothedelignan A, gymnothedelignan B	SE, CC, X-ray, NMR, MS	[97]
Selaginellaceae	*Selaginella moellendorffii* Hieron.	Whole plant	moellenoside B	SE, CC, LC, TLC, [α]_D_, CD, UV, IR, NMR, MS	[98]
Schisandraceae	*Schisandra bicolor* W.C.Cheng.	Fruits	marphenol C, marphenol D, marphenol E, marphenol F	SE, CC, LC, HPLC, [α]_D_, UV, IR, NMR, MS	[99]
Solanaceae	*Cestrum diurnum* L.	Leaves	cestrumoside, berchemol-4′-*O*-β-glucopyranoside, dehydrodiconiferyl alcohol-4-*O*-β-glucopyranoside, (+)-lyoniresinol 3a-*O*-β-glucopyranoside, (–)-lyoniresinol 3a-*O*-β-glucopyranoside	SE, CC, [α]_D_, UV, CD, IR, NMR, MS	[100]
	*Cestrum parqui* (Lam.) L’Hér.	Leaves	9′-*nor*-3′,4,4′-trihydroxy-3,5-dimethoxylign-7-eno-9,7′-lactone	SE, CC, [α]_D_, NMR, MS	[101]
	*Nicotiana tabacum* L.	Roots and stems	nicotnorlignan C, recurphenol C, recurphenol D, sequirin C, benzodioxane	n.r.	[102,103]
		Leaves	nicotnorlignan A, sequirin C, benzodioxane	n.r.	[102]
	*Solanum melongena* L.	Roots	guaiacylglycerol 8′-vanillin ether, ficusal, polystachyol	SE, CC, HPLC, [α]_D_, NMR, MS	[104]
Styracaceae	*Styrax camporum* Pohl	Whole plant	egonol, homoegonol	SE, pTLC, CC, HPLC-UV, NMR	[105]
	*Styrax ferrugineus* Nees and Mart.	Leaves	egonol, homoegonol, egonol glucoside, homoegonol glucoside	SE, FCC, IR, NMR, MS	[106]
	*Styrax japonica* Sieb. et Zucc.	Stem bark	styraxlignolide A, egonol, masutakeside I	SE, CC, LC, [α]_D_, UV, NMR, MS	[107]
	*Styrax obassis* Sieboldi and Zucc.	Aerial parts	1′′-hydroxylegonol gentiobioside, egonol glucoside	SE, CC, LC, NMR, MS	[108]
	*Styrax officinalis* L.	Fruits	egonol, dimethyl-egonol, homoegonol	SE, CC, NMR, MS	[109]
	*Styrax pohlii* A. DC.	Aerial parts	egonol, homoegonol, homoegonol gentiobioside, homoegonol glucoside, egonol gentiobioside	SE, CC, HPLC, NMR	[110]
	*Styrax ramirezii* Greenm.	Fruits	egonol, homoegonol, egonol glucoside, homoegonol glucoside, 7-demethoxy-egonol, 4-*O*-demethyl-homoegonol	SE, HPLC-DAD-MS	[111]
Thelypteridaceae	*Abacopteris penangiana* (Hook.) Ching	Rhizomes	penangianol A, penangianol B	SE, CC, [α]_D_, UV, IR, NMR, MS	[112]
Urticaceae	*Pouzolzia occidentalis* (Liebm.) Wedd.	Aerial parts	pouzolignan A, pouzolignan B	SE, CC, LC, [α]_D_, UV, IR, NMR, MS	[113]
	*Pouzolzia zeylanica* var. *microphylla* (Wedd.) Masam.	Aerial parts	pouzolignan D, pouzolignan K	n.a.	[114]

## Abbreviations

[α]_D_: Optical Rotation Spectroscopy; ECD: Electronic Circular Dichroism Spectroscopy; CC: Column Chromatography; CD: Circular Dichroism Spectroscopy; FCC: Flash Column Chromatography; HPLC: High Performance Liquid Chromatography; IM: Immunohistochemistry Methods; IR: Infrared Spectroscopy; LC: Liquid Chromatography; MP: Melting Point; MS: Mass Spectrometry; NMR: Nuclear Magnetic Resonance Spectroscopy; n.a.: not accessible; n.r.: not reported; pTLC: Performance Thin Layer Chromatography; SE: solvent extraction; TLC: Thin Layer Chromatography; UV: Ultra-Violet Spectroscopy.
